# Regional differences in fishing behavior determine whether a marine reserve network enhances fishery yield

**DOI:** 10.1038/s41598-024-51525-6

**Published:** 2024-01-12

**Authors:** Hunter S. Lenihan, Daniel C. Reed, Maria Vigo, Callie Leiphardt, Jennifer K. K. Hofmiester, Jordan P. Gallagher, Chris Voss, Peyton Moore, Robert J. Miller

**Affiliations:** 1 Marine Science Institute, University of California, Santa Barbara, CA USA; 2 Institut de Ciències del Mar, CSIC, Barcelona, Spain; 3 California Department of Fish and Wildlife, San Diego, CA USA; 4 Department of Ecology, Evolution, and Marine Biology, University of California, Santa Barbara, CA USA; 5 California Lobster and Trap Fishermen’s Association, San Marcos, USA

**Keywords:** Ecology, Environmental sciences

## Abstract

A network of marine reserves can enhance yield in depleted fisheries by protecting populations, particularly large, old spawners that supply larvae for interspersed fishing grounds. The ability of marine reserves to enhance sustainable fisheries is much less evident. We report empirical evidence of a marine reserve network improving yield regionally for a sustainable spiny lobster fishery, apparently through the spillover of adult lobsters and behavioral adaptation by the fishing fleet. Results of a Before-After, Control-Impact analysis found catch, effort, and Catch-Per-Unit Effort increased after the establishment of marine reserves in the northern region of the fishery where fishers responded by fishing intensively at reserve borders, but declined in the southern region where they vacated once productive fishing grounds. The adaptation of the northern region of the fishery may have been aided by a history of collaboration between fishers, scientists, and managers, highlighting the value of collaborative research and education programs for preparing fisheries to operate productively within a seascape that includes a large marine reserve network.

## Introduction

No-take marine reserves are effective conservation tools for protecting marine resources within reserve borders^1–5^. Protection from fishing often leads to the increased size^6^, density^5,7^, and spawning biomass^7^ of harvested species. The role marine reserves play in fishery management is less certain and widely debated. Controversy arises in part from the clear short-term costs to fishers associated with reserve implementation, which reduces the size of fishing grounds potentially leading to revenue loss, especially over the short term^8–10^. Other fishery effects of reserves include displacing and redistributing fishing effort, and influencing yield through the export, or spillover, of production from the reserve into fishable areas^11^.

Theory predicts that a network of marine reserves can stabilize or enhance fishery yield if large, old spawning individuals are protected, and the reserves are arranged in space so that unprotected areas open to fishing receive spillover from reserves^10–13^. Such spillover can be ecological (larvae, juveniles, and adults) or fishery (biomass that can be fished) in nature^14^. Tests of spillover theory using spatial population modelling indicates that larval spillover from a reserve network can enhance yield for over-capitalized fisheries that have been depleted through excess fishing^15,16^. Statistical analysis of marine reserve data also found that spillover of adult target species is a relatively common phenomenon^17^, but modelling of those data indicated that adult spillover was sufficient to sustain depleted fisheries in some cases^18,19^ and insufficient in other cases^17,19^. By contrast, results from field studies have shown that spillover of adults can enhance local catch in depleted fisheries^18,20,21^, and at least in one case a sustainable, well-managed fishery^22–24^.

Here we report the results of a Before-After Control-Impact Paired Series (BACIPS)^25^ analysis designed to test whether a large marine reserve network influenced the catch in a large, well-managed spiny lobster fishery in California, USA. Specifically, we tested the hypothesis that the establishment of a reserve network in California along the mainland coast increased total catch and catch-per-unit effort (CPUE) due to spillover of legal-sized lobsters from reserves to adjacent unprotected fishing grounds, where fishers adapted to fish near the reserve borders and intercept the emigrating lobsters. This hypothesis rested on our prediction that reserves would have no effect on total fishing effort. Our test relied on fishery-dependent catch and effort data collected by the State of California, as well as a fine-scale survey of lobster trapping effort near reserve borders.

### California’s marine reserve network

Small coastal marine reserves have existed in California since the 1930s. In 2012, the California Marine Life Protection Act (MLPA) greatly expanded the area protected from fishing by establishing a network of 86 marine reserves along California’s 1350 km of coastline^26^. Examination of the ecological effects of the MLPA reserve network revealed that recovery of exploited species of kelp forest fishes inside reserves was rapid, but highly variable in space^27^. Much less is known about the extent to which the MLPA network, or networks of marine reserves in general, have benefitted populations of fished species occurring outside of reserves.

**Figure 1:**
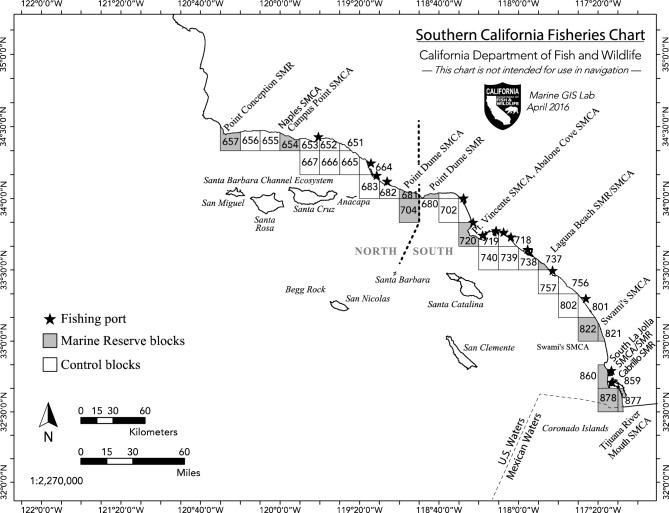
Location of the spiny lobster fishery in southern California (CA), USA. Displayed are the 12 jurisdictional fishing blocks with marine reserves (gray) and the 22 blocks without reserves (white) established in 2012. Block numbers are those used by the California Department of Fish and Wildlife to record fishery-dependent data, which for the spiny lobster fishery include the weight of lobster caught, and the number of traps pulled. Also shown are the 14 marine reserves (SMR) and State Marine Conservation Areas (SMCA) established along the coast where lobster fishing is prohibited, and the fishing ports (black stars) where fishers are based and land their catch. “North” refers to the northern region of the fishery, and “South” the southern region.

We evaluated the fishery benefits of the MLPA network along the mainland coast of southern California (Fig. 1), where the state’s most valuable reef-based fishery targets the California spiny lobster (*Panulirus interruptus*), in addition to many other commercial and recreational fisheries. Fourteen MLPA reserves established in this region in 2012 closed 10% of spiny lobster fishing grounds along the mainland coast through the protection of 137 km^2^ of ocean space, much of which was composed of lobster-rich rocky reef habitat^28^. Lobsters are also caught in shallow waters surrounding the offshore Channel Islands where other reserves established in 1974–2003 removed 17% of the fishable habitat at the islands^29^. The first and only quantitative stock assessment of the spiny lobster fishery, conducted in 2011, concluded that the fishery was sustainable^24^. The 2016 CA Spiny Lobster Fishery Management plan established an adaptive management process designed to guard against unsustainably increasing commercial and recreational fishing effort, and accounted for the MLPA marine reserves. The fishery is managed by the State of California, with no Federal or interstate councils or commissions involved.

## Results and discussion

### Effects of reserves on fishing effort and yield

**Figure 2:**
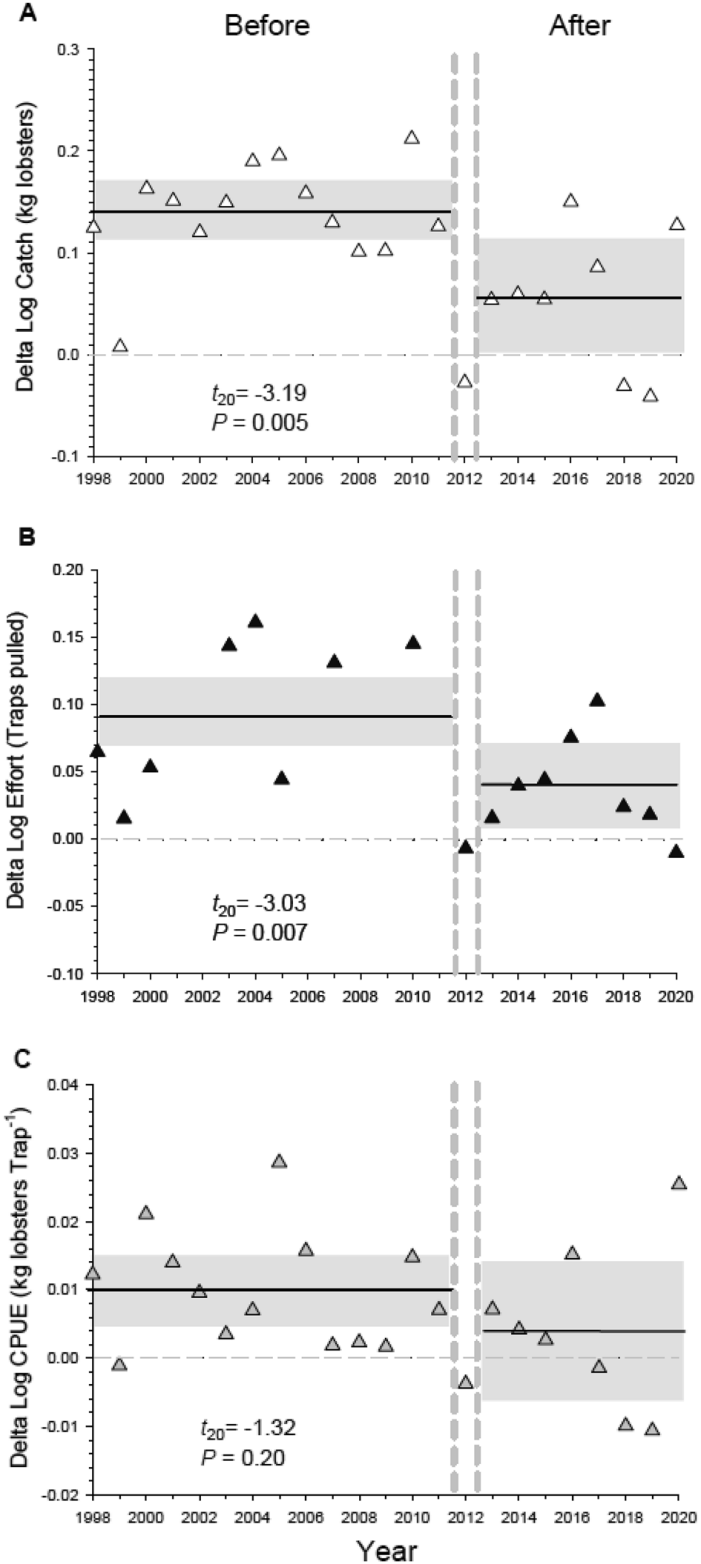
Response of the CA spiny lobster fishery to establishment of a marine reserve network along mainland coast of California, USA in 2012 (The area from Pt. Conception to the U.S.-Mexico Border in Fig. 1). Displayed are Delta values (∆ = sum of Reserve blocks − sum of Control blocks) for: (**A**) catch (kg of lobster caught per annual fishing season), (**B**) fishing effort (number of traps pulled per annual fishing season), and (**C**) the Catch-Per-Unit Effort (kg lobster caught per trap pulled per annual fishing season) for the entire fishery. Reserve blocks are jurisdictional fishing blocks with marine reserves and control blocks are fishing blocks without reserves. Fishing occurs in both block types. Horizontal lines represent the means (solid line), and the zero value (dotted line) represents no difference between the values of Reserve and Control blocks. Delta values above zero represent years when Reserve blocks had overall higher values than Control blocks. Also shown are upper and lower 95% confidence limits (grey shaded area) for the 14 years *Before*, and 8 years *After* reserves were implemented. The dashed vertical lines separate the *Before* and *After* periods, and include a transition year (2012) that was not included in the analysis (see “Methods”). Results (*P*-values) of two-sample *t*-tests comparing Delta values in the Before vs After period are shown. Data are log transformed catch, effort, and CPUE (Log Catch/Log Effort).

**Figure 3:**
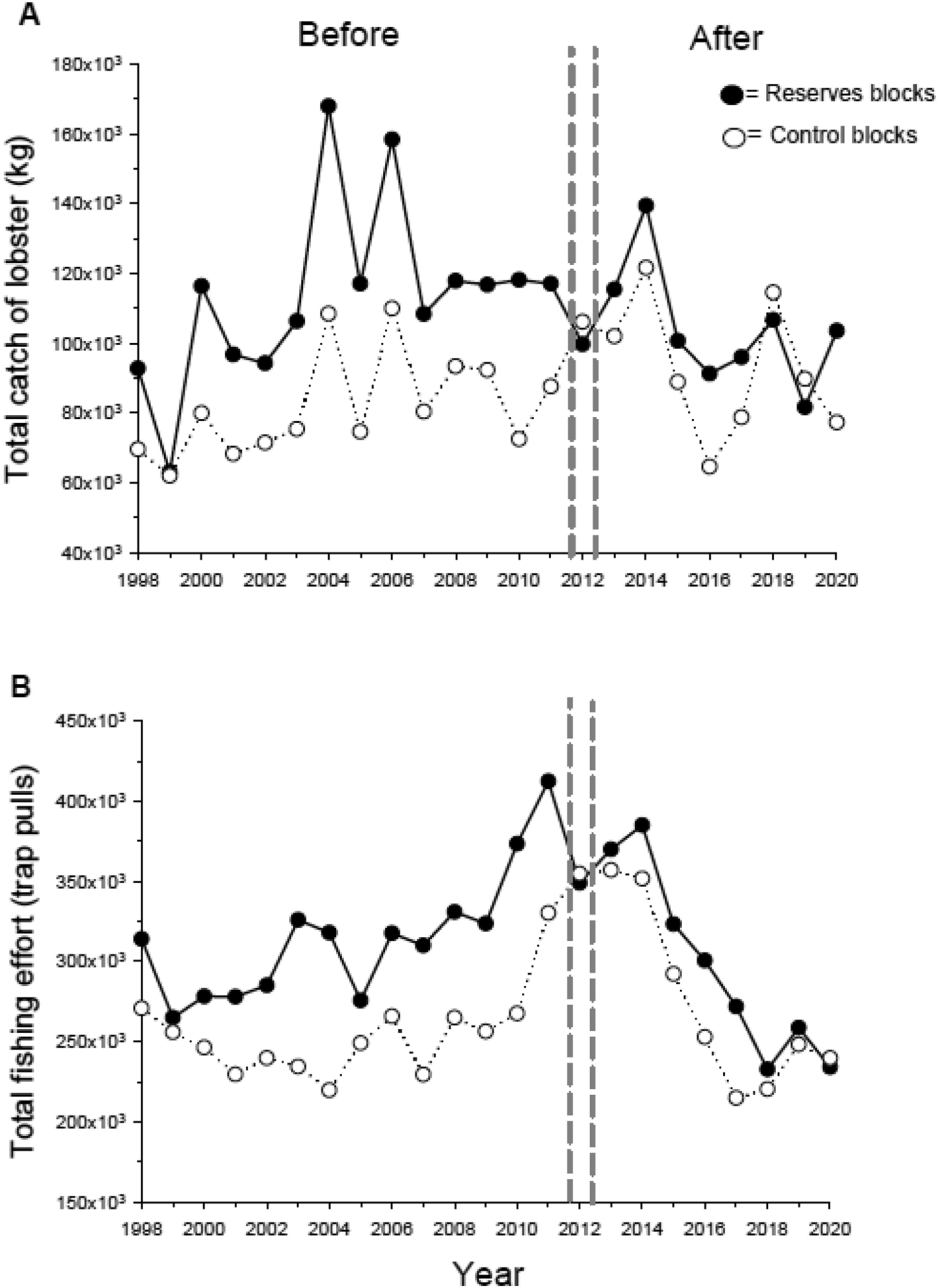
Annual summed values of the commercial mainland spiny lobster fishery in California for: (**A**) lobster catch (kg) and (**B**) fishing effort (trap pulls) for reserve (solid circles) and control (open circles) fishing blocks. These data are for the entire fishery (north and south regions combined). Reserve blocks (n = 12) are jurisdictional fishing blocks with marine reserves and control blocks (n = 22) are fishing blocks without reserves. The vertical dotted lines designate when the reserves were established and include a transitional year (2012) not used in the calculations of the Delta values shown in Figs. 2, 4, and 5.

We used data from jurisdictional fishing blocks where lobster fishing occurred and was reported from, including those with reserves, as only portions of reserve blocks were closed to fishing. Results of the BACIPS analysis showed the difference in catch (∆ = Reserve blocks—Control blocks) between fishing blocks with and without reserves was on average 13% lower in the period after reserves were established (2013–2020) than in the period before reserves were established (1998–2011; Figs. 2A, 3A). There was also a significant effect of reserves on fishing effort, effectively resulting in an average 43% decrease in the Delta log values for lobster traps set after reserves were established (Fig. 2B). Fishing effort declined overall across the entire fishery beginning in 2015 (Fig. 3B) when state fishery managers implemented a limit of 300 traps per fishing permit^24^. The number of traps set per fishing permit was not limited prior to 2015. While the implementation of a trap limit caused an overall reduction in fishing effort, it did not account for the reduced effort observed in the reserve blocks relative to the control blocks in the after period (Fig. 2B), as trap limits applied equally to reserve and control blocks. There was no significant effect of reserves on lobster CPUE, which varied substantially over time in both the before and after periods (Fig. 2C).

**Figure 4:**
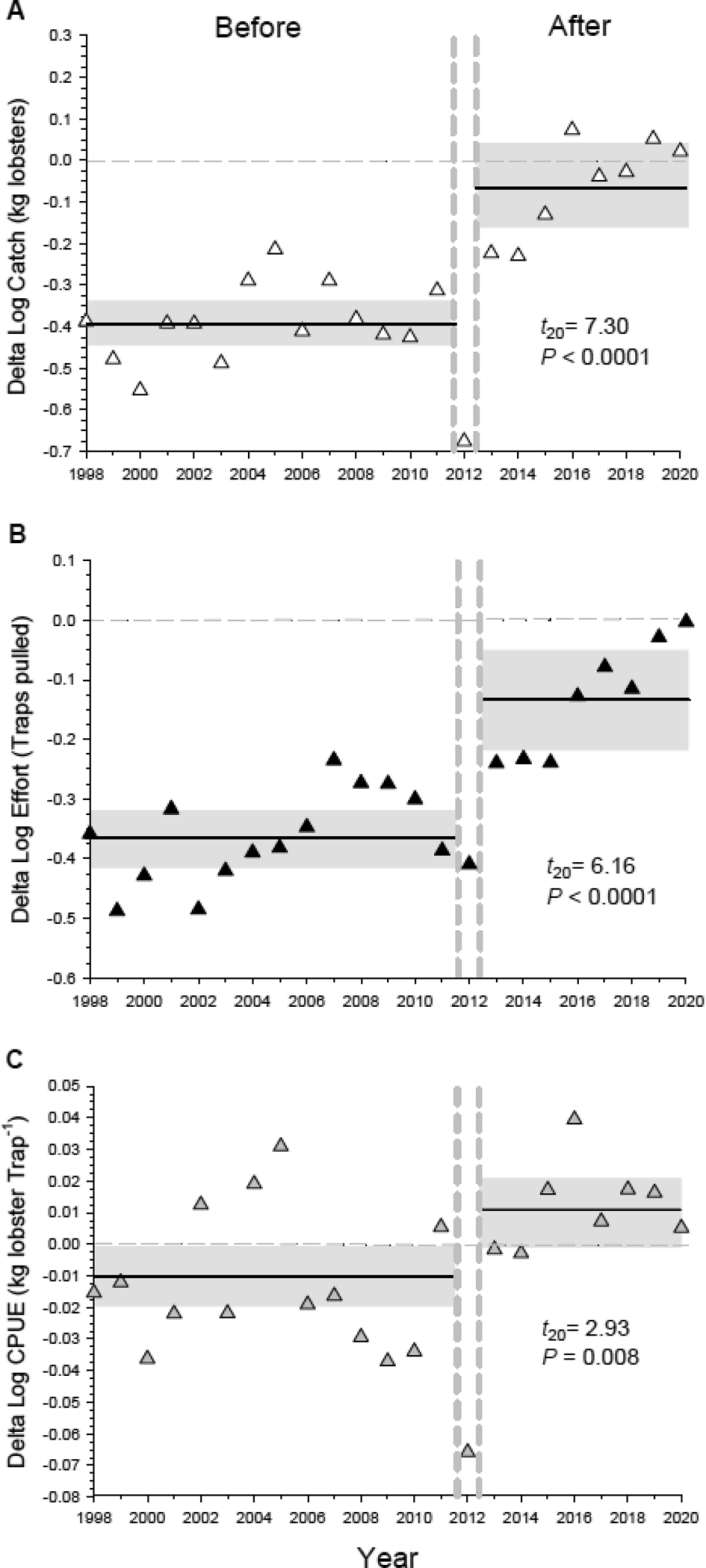
Response of the spiny lobster fishery to the establishment of a marine reserve network in the northern portion of the fishery (“North” in Fig. 1). Displayed are log transformed Delta values for (**A**) catch, (**B**) effort, and (**C**) CPUE (Log Catch/Log Effort). See Fig. 2 for definitions of catch, effort, CPUE and the horizontal and vertical lines. Results (*P*-values) of two-sample *t*-tests comparing delta values in the Before vs After period are shown. Untransformed values for catch, effort and CPUE are displayed in Fig. S2.

A closer examination of the data revealed dramatic regional differences in the effect of reserves on the fishery. In the northern region (Point Conception to Point Dume; Fig. 1) the Delta values for catch increased threefold after the establishment of reserves while effort and CPUE doubled (Fig. 4A–C). This pattern emerged even though effort in control blocks without reserves during the after period was as high or higher than that in the before period (Fig. S1), indicating the positive effects of reserves on lobster catch and CPUE in this region. The switch from negative Delta values of CPUE in the before period to positive values in the after period (Fig. 4C) further signifies that reserves had a substantially positive overall effect on the fishery in the northern region. We reason that increased effort in reserve blocks in the after period benefited the fishery through increased catch because there were more lobsters to catch via spillover. We documented a similar pattern at a local level in prior work^22,23^.

**Figure 5:**
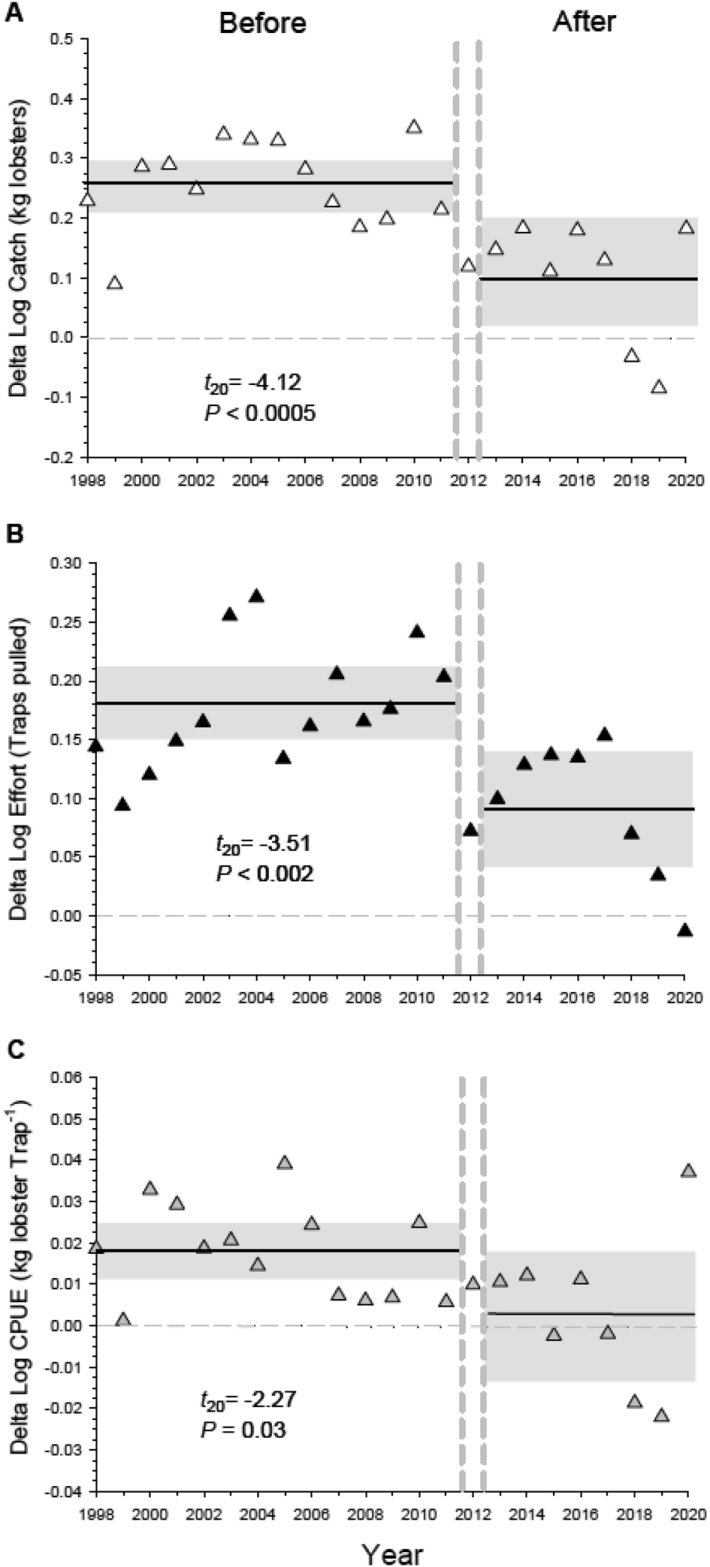
Response of the spiny lobster fishery to the establishment of a marine reserve network in the southern portion of the fishery (“South” in Fig. 1). Displayed are log transformed Delta values for (**A**) catch, (**B**) effort, and (**C**) CPUE (Log Catch/Log Effort). See Fig. 2 for definitions of catch, effort, CPUE and the horizontal and vertical lines. Results (*P*-values) of two-sample *t*-tests comparing Delta values in the Before vs After period are shown. Untransformed values for catch, effort and CPUE are displayed in Fig. S3. Please note the difference in the scale on the Y-axis between this Figure and Fig. 4.

By contrast, in the southern region (Pt. Dume to Mexico) Delta values for catch, effort, and CPUE decreased significantly after the establishment of reserves (Fig. 5A–C), thus driving the negative effect of reserves at the entire fishery level (Fig. 2A). Overall effort in the southern region, and to a lesser extent catch, declined in both the reserve and control blocks after reserve establishment (Fig. S2), and the negative effect of reserves on CPUE (Fig. 5C) reflects a greater decline in effort in reserve blocks relative to control blocks (Fig. S2B). It should be noted that total lobster fishing effort and catch in the southern region is consistently three to four times higher than the northern region (Figs. S1, S2). These differences are due largely to the larger size of the southern region, which has approximately four times more fishable area and three times as many fishing ports, thus supporting about three times as many fishers compared to the northern region^24^.

### Regional differences in fishing behavior

**Figure 6:**
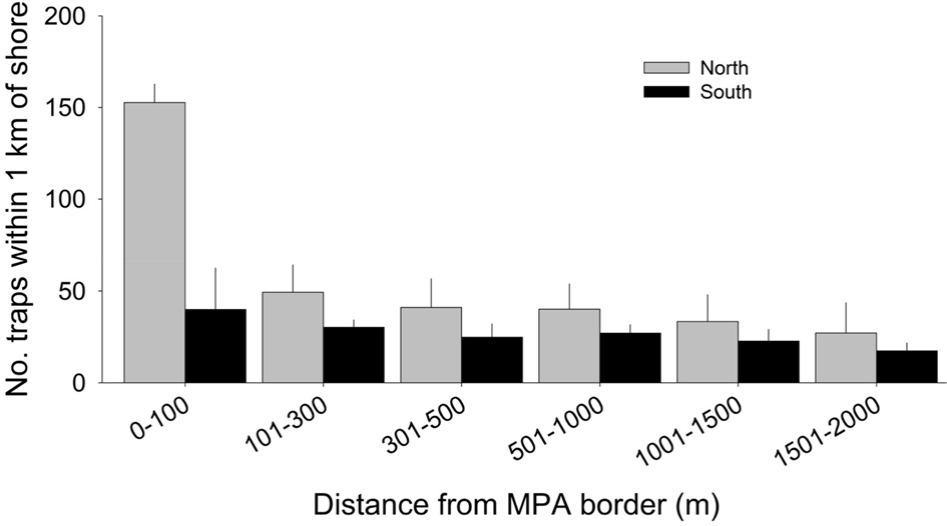
The mean number (± SE) of lobster traps placed at increasing distances from borders of three reserves in the northern portion of the fishery (Point Conception, Campus Point, and Point Dume) and three reserves in the southern region (Point Vincente SMCA, Matlahuay, and South La Jolla). There were more traps in the northern region (gray bars) than the southern region (black bars) (*P* < 0.05) within 2 km of reserves, despite there being many more traps set in the south than north (See “Results and discussion” for details).

Our prior work showed increases in spiny lobster abundance within two reserves located in one fishing block in the northern region, and the related spillover of legal-sized adult lobsters, as well as enhanced lobster catch and CPUE in that fishing block relative to nearby blocks without reserves^22^. Prior research also revealed that lobster fishers often concentrate their trapping effort near reserve borders^22,30,31^, in part due to the fisher’s participation in collaborative research with scientists and awareness of its results^23,32^. We reasoned that differences in lobster yield between the north and south regions associated with the establishment of the MLPA reserves could be explained by differences in fishing behavior between the two regions, specifically the degree to which fishers in each region fished near reserve borders early in the fishing season (October–November) when landings are by far the highest. Evidence for this comes from our field observations of trapping effort in which we recorded a much higher average number of traps within 2 km of the borders reserves in the north than we did in the south (Fig. 6; *F*_5,35_ = 12.29; *P* < 0.002), despite a far greater number of traps set overall in the south (Fig. S2B vs. Fig. S1B). This result indicated that fishing reserve borders, relatively early in the season, is more prevalent and intensive in the north than the south.

Of course, factors other than fishing behavior may help explain the spatial difference in catch in response to reserve establishment. For example, historical logbook catch data indicate that reserves in the south were placed in relatively more productive lobster fishing blocks than reserves in the north (see Figs. S1A and S2A), suggesting that the quality and carrying capacity of reef habitat may vary between the two regions. Such habitat differences could influence lobster movement out of reserves^32,33^ and thus spillover^34^.

Our research team included a CA spiny lobster fisherman with three decades of fishing experience, a state resource manager, and scientists who began collaborating on research with the lobster fishery in 2002^23,32^. Our collective experience and conversations with fishers lead us to believe that regional differences in the effects of reserves were generated by interactions of fleet dynamics, fishing behavior, and the physical environment. We learned that, in response to reserve establishment in 2012, some of the most productive fishers who fished in reserve areas in the south moved their fishing effort to the northern region, where there are fewer fishers and less competition for space^28,29^. This helps to explain the reduction of fishing effort in reserve blocks in the south (Fig. S2B) and the increased effort in reserve blocks in the north (Fig. S1B) following reserve establishment.

In our collaborative research with lobster fishers^32^ we discovered that baited traps are generally set in groups or lines located along edges of rocky reef habitat, where lobsters normally forage for food and find refuge from predators and physical disturbance caused ocean swell. Spiny lobsters’ attraction to the baited traps apparently increases when competition for food and refuge intensifies, for example, within or adjacent to reserves where population abundances are significantly higher due to protection^35^. Lobsters also move from shallow rocky habitat to deeper water as wave intensity increases throughout the fishing season (October–March). Fishers in turn shift their traps from shallow to deep water to avoid trap loss or damage caused by swell, and to intercept lobsters that are migrating from shallow to deep water. This shift in fishing behavior typically involves placing lines of traps near rocky habitat located at increasing depths as the season progresses. For some fishers this includes shifting traps to areas with rocky habitat located near reserve borders. We also learned that fishers in the north generally move their traps into deeper water earlier in the season than those in the south because swell size increases earlier in the north than south (*Personal Communication* with fisher S. Escobar, who has fished both regions). Accordingly, northern fishers may move their traps to reserve borders earlier in the season, when lobster catch is relatively high, than fishers in the south. Other potential causes of shifts in fishing behavior, such as fishers exiting the fishery or changes in dockside value, are unlikely to have led to these shifts, based on discussions with fishers and resource managers (*Personal communication*).

Regional differences in fishing behavior that altered the effectiveness of reserves on fishery yield may also reflect cultural differences in addition to environmental differences. Beginning in 2002, we developed a collaborative fishery research program, (*CALobster*) in the northern region of the fishery to assess the effects of marine reserves on lobster populations, spillover, and fishery yield^22,23,32^. Our collaboration relied on research trapping campaigns with fishers as well as numerous formal and informal meetings with members of the California Lobster and Trap Fisherman’s Association (CLTFA) and the CA Department of Fish and Wildlife (CDFW), at which our results pertaining to the responses of lobster populations to reserve protection were discussed. This concerted and sustained effort to share information increased understanding among stakeholders about the predicted and actual effects of the MLPA reserves on lobsters and fishers^32^, and stimulated an increase in the propensity of the fleet to fish the border of reserves in the north (C. Voss, *personal observations*). Similar types of collaborations and interactions have enhanced the performance of fisheries in other regions in response to reserve establishment^36,37^.

Collectively, our results demonstrate that a reserve network can have an overall positive influence on lobster catch, even in a well-managed sustainable fishery, where fishers respond to spillover of target species from reserve borders. Understanding the primary factors determining population and behavioral responses of targeted species to reserve establishment remains difficult, but communicating research results on these responses can assist fishers to adapt to reserve establishment. Collaborative marine reserve research involving diverse stakeholders helps scientists to better understand conservation actions on human livelihoods, while also helping fishers to operate productively within seascapes that include large marine reserve networks^38^. Our results suggests that the fishery benefits of marine reserves will be enhanced when collaborative research involving diverse stakeholders and Before-After assessments are incorporated into marine reserve design and implementation.

## Materials and methods

### Fishery-dependent data and analysis

#### The fishery

The commercial fishery for spiny lobster in California, USA extends from Morro Bay (35.3659°N 120.8500°W) south to the US–Mexico border and involves fishers using relatively small boats to deploy baited wire box-like traps set on the bottom in shallow reef habitats. Traps are set in water depths from 3 to 170 m. The fishing season is from October to March with approximately 80% of the annual catch landed within the first half of the season. It is assumed that most of lobsters landed by the fishery reached legal size (83 mm carapace length) during the previous year, but this has yet to be confirmed.

#### Fishing blocks and recorded catch

California (CA) fisheries are managed by the CA Department of Fish and Wildlife (CDFW), who has divided the entire coastline into rectangular fishing blocks (~ 140 km^2^), from which commercial lobster fishers, and other fisheries, are required to record and log all their landings and fishing effort (https://nrm.dfg.ca.gov/FileHandler.ashx?DocumentID=67449&inline). Annual fishing block data of commercial lobster landings (wet pounds caught) and fishing effort (number of traps pulled) were obtained from CDFW for all fishing seasons for which data were recorded (1998–2020). We defined a fishing season by the year in which it started (e.g., the 2012 season extended from October 2012 through March 2013). Fishing block data are based on landings weighed at the dock by the processor, who records the data on a “fish ticket” that is submitted to the CDFW. Fishers are required to assign their landings to a specific fishing block (recorded in “fish ticket” data) and report the fishing effort (i.e., number of traps pulled; recorded in fisher logbooks) allocated to their catch. Although Catch-Per-Unit-Effort (CPUE) is defined by CDFW as the number of legal lobsters per trap pull, for our analyses we defined it as the weight of legal lobsters caught per traps pulled.

### Designation of fishing blocks

The study region included commercial fishing blocks that extend along the mainland coast of California (Fig. 1). The southern California lobster fishing fleet is organized primarily around ports where fishing boats are moored and fishers land their catch. Through our ongoing collaborative research program, we learned that a majority of boats fishing within the northern section of the fishery utilized the four northern ports (Santa Barbara, Channel Islands, Ventura, and Hueneme) located in Ventura and Santa Barbara counties. Many lobster fishers using these ports were active members of the California Lobster and Trap Fishermen’s Association, a social organization with whom we shared information and developed research projects over the past two decades. Fishers from the southern region of fishery, who fished south of Pt. Dume to the US-Mexico Border, utilized mainly 10 ports (Marine del Rey, King, Los Angeles, Alamitos, Sunset-Huntington, Newport, Dana Point, Oceanside, Mission Bay, and San Diego) located in Los Angeles, Orange, and San Diego counties. We had very few interactions with fishers from these ports.

Thirty-four fishing blocks located along the coastline where MLPA reserves were established in 2012 reported spiny lobster commercial fishing data to the CDFW (Block #s: 651, 652, 653, 654, 655, 656, 657, 664, 665, 666, 667, 680, 681, 682, 683, 702, 704, 718, 719, 720, 737, 738, 739, 740, 756, 757, 801, 802, 821, 822, 859, 860, 877, 878). Fourteen State Marine Reserves (SMR) or State Marine Conservation Areas (SMCA) that did not allow lobster fishing were established in 12 of those blocks in 2012 (Blocks #657—Point Conception SMR; 654—Naples/Campus Point SMCA; 681 and 704—Pt. Dume SMCA; 680—Pt. Dume SMR; 720—Pt. Vincente SMCA and Abalone Cove SMCA; 737—Laguna Beach SMCA/SMR; 821 and 822—Swami’s SMCA; 860—South La Jolla SMCA/SMR; 877—Tijuana River Mouth SMCA; and 878—Cabrillo SMR). Four of the no-lobster fishing blocks were in the northern region (654, 657, 681, and 704; Fig. 1); while 8 reserve blocks (680, 720, 737, 821, 822, 860, 877, and 878) were in the southern region. The remaining 22 blocks where lobster catch was recorded were control blocks without reserves, or were blocks with SMCAs that allowed lobster fishing (block 656 in the northern region with Kashtiyat SMCA; and blocks 738 and 757 in the southern region with Crystal Cove and Dana Point SMCAs). The distribution of control and reserve blocks in the different regions of the fishery are illustrated in Fig. 1.

### Statistical analysis

The effects of fishing status (blocks with reserves versus blocks without reserves), and period (i.e., before versus after MLPA implementation) on annual catch, effort, and catch-per-unit-effort (CPUE) were evaluated using a Before-After, Control-Impact, Paired Series (BACIPS) design^25^ in which Delta values were calculated as the difference Reserve blocks—Control blocks, for each of the 14 annual fishing seasons “Before” MLPA implementation (1998–2011) and eight seasons ”After” implementation (2013–2020). We removed the 2012 season when reserves were first activated from our analyses because many fishers were forced to re-establish new fishing grounds in the first year after reserve establishment leading to overall less intensive fishing^29^. A Delta value for each response variable was calculated for each season by subtracting the total value summed from the control blocks from the total sum from reserve blocks. The population of Deltas in the before period (N = 14) were then compared with that from the after period (N = 8) in a two-sample T-test with equal variances, as verified by a folded F-test. Separate BACIPS analyses were conducted for the entire fishery, the northern region of the fishery (Pt. Conception to Pt. Dume) and the southern region (Pt. Dume to the US-Mexico Border). Before the analyses, catch and effort data were log transformed to meet the assumptions of additivity in the BACIPS analysis. CPUE was calculated as the log catch/log effort. All statistical analyses were conducted in SAS software.

### Lobster fishing behavior

Fishing behavior in the vicinity of the no-fishing zones was examined by counting lobster trap buoys at increasing increments of distance (0–100 m, 101–300 m, 301–500 m, 501–1000 m, and 1001–1500 m) away from reserve borders visually from land with a spotting scope. Each trap buoy is connected to one lobster trap. Buoys within 1 km of shore were counted from shore, in each distance increment. We reported as the number of traps within 1 km of shore because all reserve borders began at the shoreline. Three no-fishing zones in the northern region (Pt. Conception SMR, Campus Point SMCA, and Pt. Dume SMR/SMCA) and southern region (Pt. Vincente SMCA, Matlahuayl SMR, and South La Jolla SMR/SMCA) were surveyed for buoys in November 2021 over a 2-day period of excellent fishing conditions. We focused our observations on these six reserves because they were close enough to shore for us to accurately count traps. These reserves were considered representative of many other MLPA reserves because of their proximity to shore, size, and quantity of lobster habitat. We used a similar method in our previous study of the Campus Point SMCA^22^.

### Statistical analysis

Mean differences in the number of traps among distance increments between the three reserves in the north and south regions were analyzed with a Two-Way Analysis of Variance (ANOVA), in which Region (north vs. south) and Distance interval were fixed factors, and Region × Distance was the interaction. The Tukey test was used for the post-hoc analysis. Data were log transformed prior to analysis to meet the assumptions of normality and homoscedasticity of ANOVA.

### Percentage of lobster fishing grounds inside versus outside of marine reserve

The area of lobster fishing grounds removed within the no-fishing marine reserves reported in the text was calculated by overlaying the CDFW fishing blocks (https://wildlife.ca.gov/Conservation/Marine/GIS/Downloads) with no-take reserves and bathymetry data provided by GEBCO (https://www.gebco.net/data_and_products/gridded_bathymetry_data/). We calculated the total fishable area within each fishing block by first confining the blocks to the deepest depth at which a lobster trap was recorded being placed (79 m). There are records of traps being set in water as deep as 170 m, but the vast majority were set in < 80 m depth. This layer was then overlaid with the boundaries of no-take reserves that were restricted to the same depth, and the restricted fishable area and the remaining area of fishing grounds were estimated. All spatial analysis was performed in R (R Core Team 2019) using the packages 'raster'^39^, 'maptools'^40^, and 'rgdal'^39^. Those data are reported in the text.

### Supplementary Information

Supplementary Figures.

## Data Availability

The raw datasets, specifically of spiny lobster fishery Logbook data and Fish Ticket data, analyzed for this study are not publicly available due to legally mandated confidentially as specified by State of California Fish and Game code 8022. They are available from the California Department of Fish and Wildlife on reasonable request. Requests for the data should be made to the California Marine Region Office (Region 7) using email address *R7MarineData@wildlife.ca.gov*, or by calling the Region 7 office at 001-831-649-2870.
