# Supplementary material for: Regional differences in fishing behavior determine whether a marine reserve network enhances fishery yield
Source: Sci Rep. 2024 Jan 12;14:1242. doi: 10.1038/s41598-024-51525-6 (PMC10786943; doi:10.1038/s41598-024-51525-6)
Supplement: Supplementary file 1 — Supplementary Figures. [file 41598_2024_51525_MOESM1_ESM.pdf]

Supplemental information: extended data

Regional differences in fishing behavior determine whether a marine reserve network enhances fishery yield

Hunter S. Lenihan[1], Daniel C. Reed[1], Maria Vigo[2], Callie Leiphardt[1], Jennifer K.K. Hofmiester[3], Jordan P. Gallagher[4], Chris Voss[5], Peyton Moore[1], and Robert J. Miller[1]

[1]Marine Science Institute, University of California, Santa Barbara, California, USA

[2]Institut de Ciències del Mar, CSIC, Barcelona, Spain

[3]California Department of Fish and Wildlife, San Diego, CA

[4]Department of Ecology, Evolution, and Marine Bioilogy, University of California, Santa Barbara, California, USA

[5]California Lobster and Trap Fishermen's Association

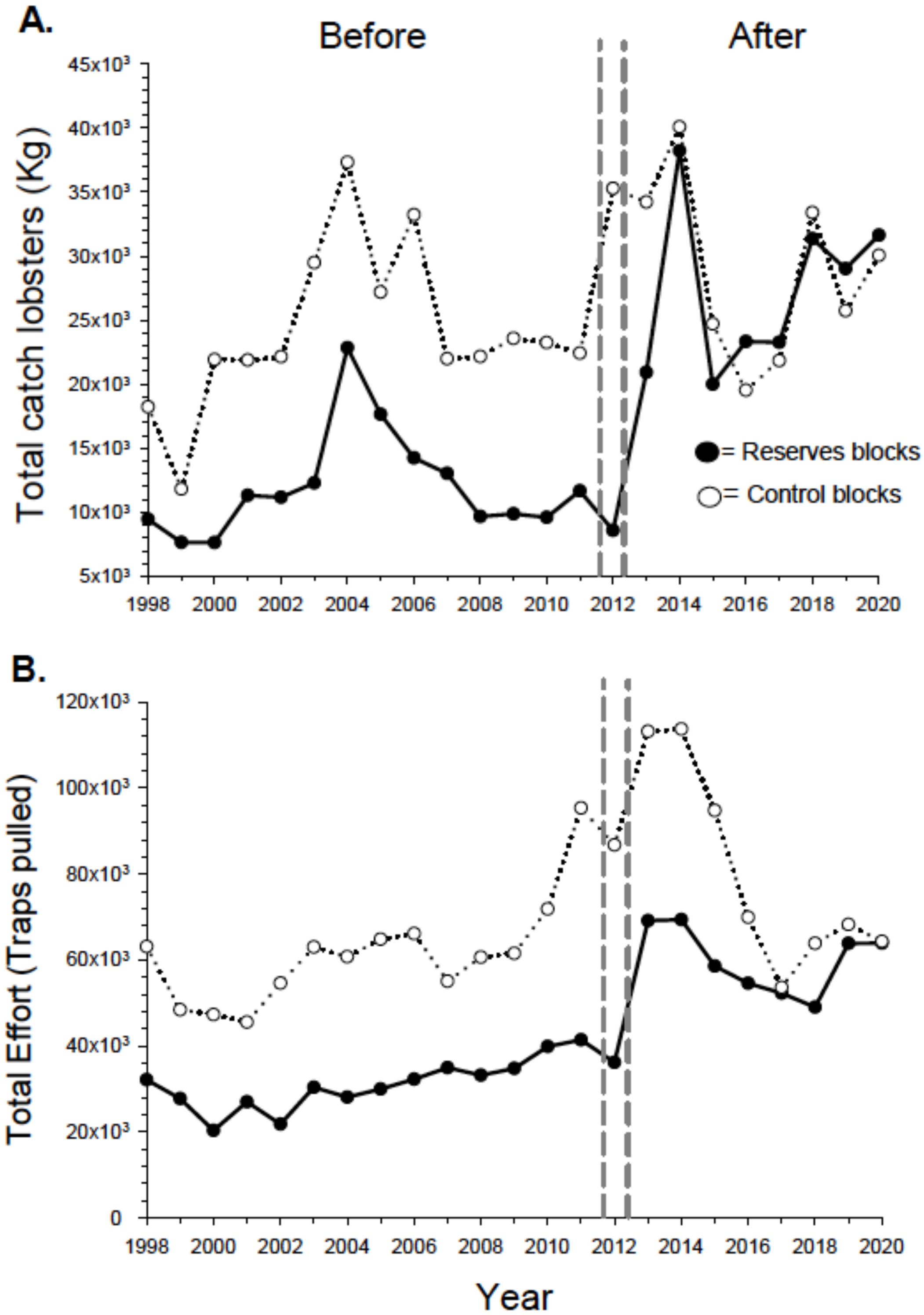

Figure S1. Annual summed values of the commercial mainland spiny lobster fishery in northern portion of the fishery (See Figure 1) for (A) lobster catch (kg) and (B) effort

(trap pulls) for fishing blocks with reserves (n = 4) and fishing blocks without reserves (control blocks, n = 11).

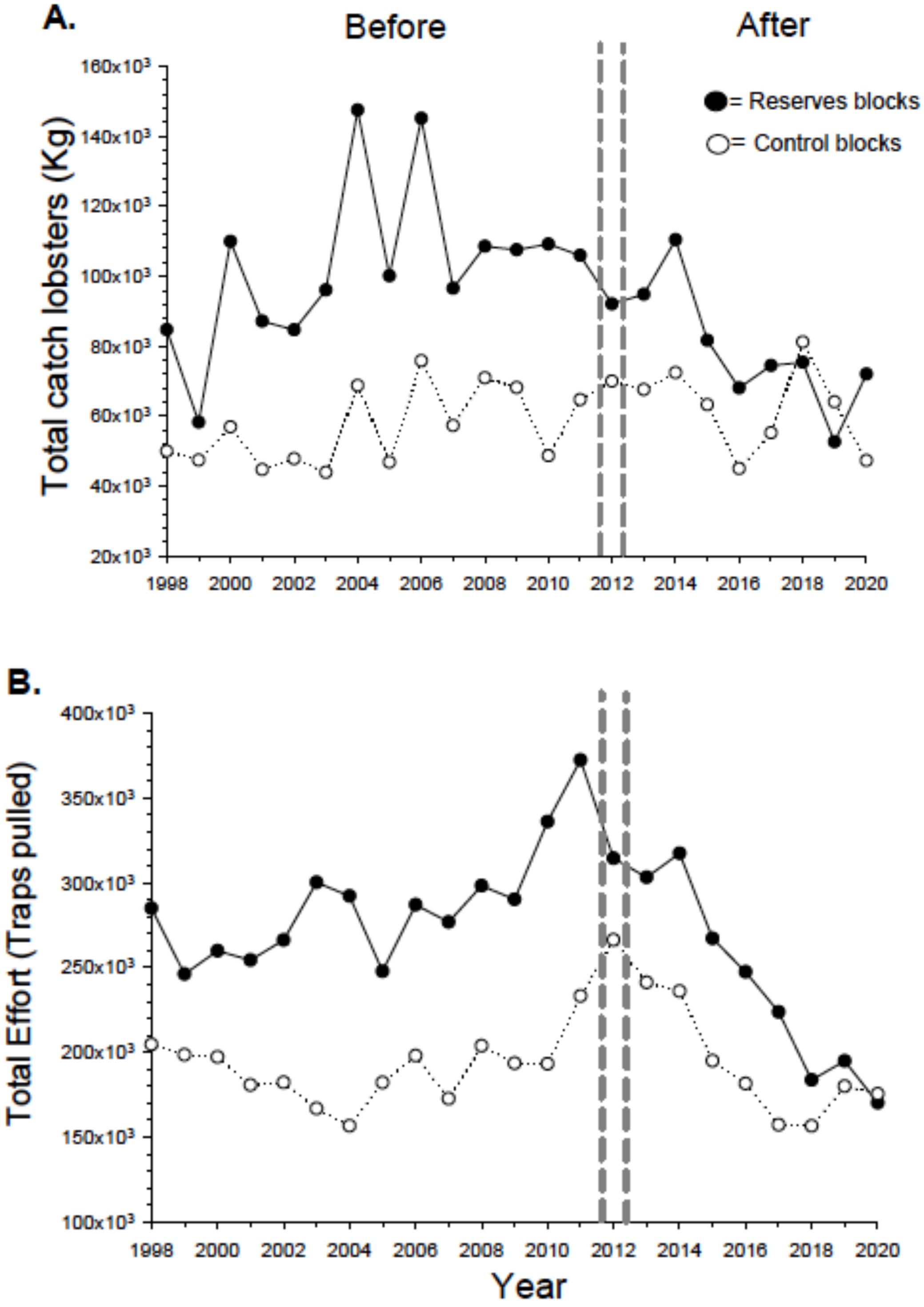

Figure S2. Annual summed values of the commercial mainland spiny lobster fishery in southern portion of the fishery (see Figure 1) for (A) lobster catch (kg) and (B) effort

(trap pulls) for fishing blocks with reserves (n = 8) and fishing blocks without reserves (control blocks, n = 11).
